# Epidemiology of penicillin allergy labels in a general Swedish population

**DOI:** 10.1016/j.jacig.2026.100761

**Published:** 2026-07-17

**Authors:** Ágnes Csuth, Maria C. Jenmalm, Lene H. Garvey

**Affiliations:** a Division of Inflammation and Infection, Department of Biomedical and Clinical Sciences, Linköping University, Linköping, Sweden; b Clinical Department of Allergy Center in Linköping, Region Östergötland, Linköping, Sweden; c Allergy Clinic, Department of Dermatology and Allergy, Copenhagen University Hospital-Herlev and Gentofte, Copenhagen, Denmark; d Department of Clinical Medicine, University of Copenhagen, Copenhagen, Denmark

**Keywords:** Penicillin allergy, delabeling, prevalence, multidrug-resistant bacteria, epidemiology, drug allergy

## Abstract

**Background:**

Penicillin allergy prevalence is reported in up to 10% of patients and is linked to broad-spectrum antibiotic overuse, extended hospitalizations, and higher mortality. Its prevalence has not previously been evaluated in Sweden.

**Objective:**

The aim of this study was to investigate the prevalence and characteristics of penicillin allergy labels (PALs) in the electronic medical records of 3 Swedish regions (1,088,000 inhabitants).

**Methods:**

A retrospective analysis of PALs in the electronic medical records in the Southeast Healthcare Region of Sweden and estimation of point prevalence were performed. Two nonoverlapping cohorts were defined by label complexity: a simple cohort with pre-2017, nonstandardized labels identified by keywords (n = 7,514), and a complex cohort (n = 22,736) with post-2017, standardized labels identified using Anatomical Therapeutic Chemical codes.

**Results:**

Point prevalence of PALs was 2.8% across all regions (69.6% female). Differences were noted across the 3 regions for the different phenotypes. The regional prevalences of skin symptoms were 15.9-32.2% for urticaria and angioedema, 2.4-23.8% for maculopapular or undefined rash, and 4.9-7.6% for flushing. Severe reactions were rare (3.0-6.9%). Mild nonallergic symptoms appeared in 1.9-2.0%, with unclear symptoms in 24.4-64.7%. The most prevalent culprits were narrow-spectrum penicillins (32.9-48.1%). Multidrug-resistant bacteria were significantly more prevalent in all regions among patients with a PAL (Östergötland, *P* = .03, Jönköping, *P* < .001 and Kalmar, *P* = .049).

**Conclusion:**

Overall, 2.8% of the study population carried a PAL. Labels need to be better documented: Up to two thirds lacked sufficient information to determine the phenotype and other characteristics of the reactions. Moreover, antimicrobial resistance was more prevalent among patients with a PAL.

Penicillin allergy (PA) is vastly overdiagnosed, and the prevalence of allergy labels or self-reported PA varies in different countries and cohorts. In affluent countries, PA prevalence is reported to be as high as 10%, yet up to 90% of PA labels (PAL) can be removed after a drug allergy assessment, including drug challenge.^1^^,^^2^ PA delabeling has a positive effect on the use of antibiotics as the use of penicillin has been shown to increase after delabeling.^3^

Different prevalences of PALs are reported in different populations and regions. Specifically, in Scandinavia, a prevalence of 4.6% was observed in an internal medical clinic in Norway, whereas a 10% prevalence was noted among hospitalized patients in Denmark.^4^^,^^5^ Epidemiologic studies of PALs in general populations are relatively scarce. A 4.5% prevalence of self-reported allergy to β-lactam antibiotics was documented in Porto, Portugal,^6^ and a meta-analysis conducted by Sousa-Pinto et al revealed that 41% of all drug allergy labels stem from self-reported penicillin allergies.^7^ Additionally, a Dutch study revealed that the prevalence of PALs was 0.6% in an extended primary care population, and that carrying a PAL was associated with increased antibiotic use and more health care use.^8^

A PAL is associated with a wide range of negative outcomes, including the overuse of broad-spectrum antibiotics, increased antimicrobial resistance, prolonged hospital stays, and higher mortality rates.^9^, ^10^, ^11^ Epidemiologic studies indicate that carrying a PAL increases the risk of carrying labels for related and nonrelated antibiotics, and that PALs are more prevalent among women and the elderly.^12^

The prevalence of reported PA has not previously been evaluated in larger geographic areas in Sweden, which is internationally recognized for its antibiotic stewardship. Narrow-spectrum antibiotics, primarily penicillins, are first-line therapy for most community-acquired infections, and antibiotic policies in primary care in Sweden are restrictive.^13^^,^^14^ In line with national recommendations, a substantial shift has taken place from broad-spectrum to narrow-spectrum antibiotics in outpatient and hospital care settings.^15^

The aims of this study were to (1) determine the point prevalence of PALs recorded in the electronic medical records (EMRs) within the Southeast Healthcare Region of Sweden, (2) to analyze recorded symptoms and suspected culprits, and (3) to determine the prevalence of multidrug-resistant bacteria, bloodborne diseases, and nonpenicillin drug allergy labels in patients with PAL compared to control groups.

## Methods

The point prevalence of PALs was investigated across 3 Swedish regions: Östergötland, Jönköping, and Kalmar, with a total population of 1,088,000 individuals. The study was approved, with a waiver of consent, by the regional ethics committee (Dnr 2023-02447-01), and the local ethics committees of the respective regions. Only allergy labels were reviewed in this study; there was no access to the complete EMR. The allergy labels extended from the 1980s, when only paper-based records were used, to March 2024. EMRs were introduced successively from 2006 to 2010. A software update in 2017 enabled caregivers to document suspected or diagnosed drug allergies in a more detailed way, which led to changes in the labels.

Systematic searches to gather data on individuals with PAL were conducted by IT technicians employed by the regions responsible for managing the EMRs. The search was based on keywords or Anatomical Therapeutic Chemical (ATC) codes, which resulted in an overly broad data capture. Therefore, a subsequent manual review by an allergist was required to remove irrelevant entries, such as APC resistance or PCI, which were included in the original search because they contained the letters “PC.” No labels indicative of PA were removed manually.

Inclusion criteria were having a current PAL with a relevant ATC code or recorded as free text. Deceased individuals were removed. Detailed methods are provided in the Online Repository available at www.jaci-global.org.

Before 2017, suspected drug allergies were recorded in free text, resulting in simple nonstandardized labels. After a software update in 2017 in all 3 regions, standardized information requirements were introduced, adding complexity by including fields for symptoms, level of suspicion, severity grade, and ATC codes. The exact dates of the cohort reviews are unknown. The new program that enabled the caregivers to register the complex labels was introduced during fall 2017 across the health care region. Labels began successively to be revised after the introduction of the program. Medical records in all 3 regions carried a color-coded symbol indicating drug allergies, multidrug-resistant bacteria, bloodborne infections, food allergies, and care warning. The different sections indicated areas where caution was necessary and were independent from each other. This enabled health care personnel to capture information about other conditions than just allergy.

We identified two distinct, nonoverlapping cohorts on the basis of label complexity: a simple cohort with nonupdated labels from before 2017, and a complex cohort with standardized labels updated or recorded after 2017. Because simple cohorts were identified by using keywords, they required manual review to remove entries not related to PA to enhance specificity, whereas complex cohorts were identified using ATC codes, which resulted in more accurate identification.

In Östergötland and Kalmar, both a simple and a complex cohort were identified (nonoverlapping), whereas in Jönköping, all labels were updated in 2017, so only a complex cohort was established (Table I). Control groups consisted of randomly selected individuals without a PAL, matched 1:1 to the complex cohorts for age, sex, and municipality. However, the match was not strictly 1:1 as a result of the removal of deceased individuals. Within the control groups, registry data were used to determine the presence of multidrug-resistant bacteria and bloodborne diseases. The time interval between prescription and entering the PAL was not available. The dataset in this study was collected at 3 time points between June 2023 and March 2024. Because the complexity of the labels differed and the data were aggregated in certain cohorts, we were able to analyze the variables with varying degrees of depth.

**Table I:** Accessible variables according to Southeast Healthcare Region of Sweden

Characteristic	Region		
	Östergötland	Jönköping	Kalmar
Cohorts (no. of labels)	Simple and complex (5,720 + 8,225)	Complex (6,664)	Simple and complex (1,794 + 7,847)
Sex	Available in total cohort	Available in total cohort	Available in total cohort
Culprit	Available in simple cohort only	Available in total cohort	Available in total cohort
Age	Exact ages are available in total cohort	Age groups are available in total cohort	Age groups are available in total cohort
Registration date for labels	Not available	Available after 2017 (only year)	Not available
Caregivers who entered labels	Available in complex cohort only	Available in total cohort	Available in complex cohort only

Details on the methods—including EMR, identification of the cohorts and control groups, and documentation of symptoms—can be found in the Online Repository. Table I lists the variables that were accessible in the different regions.

Statistical analysis was conducted by Excel 2016 and IBM SPSS v29.0.1.1 software. Descriptive statistical analysis was performed to ascertain the frequency distribution of the symptoms, age groups, culprits, age demographics, and the clinics that recorded the labels. The prevalence rates of multidrug-resistant bacteria, bloodborne diseases, and nonpenicillin drug allergies between the complex cohorts and control groups were compared by the chi-square test, with *P* < .05 considered statistically significant. Risk ratios and 95% confidence intervals were calculated for each comparison of prevalences.

## Results

**Fig 1:**
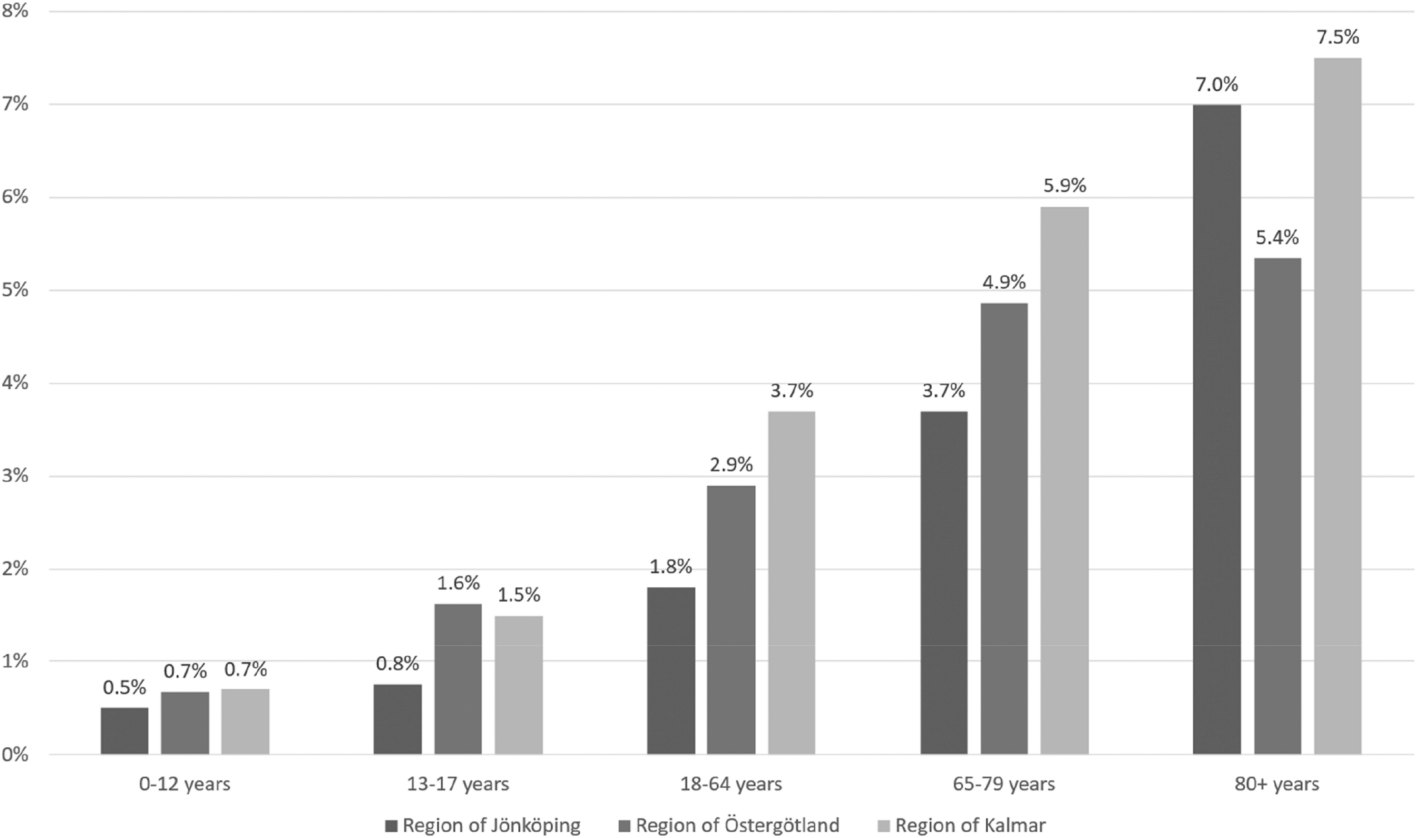
Prevalence of PALs in different age groups across regions of Southeast Healthcare Area of Sweden.

The point prevalence of PALs was 2.8% for the total cohort (n = 30,250) in the Southeast Healthcare Region; the majority were female (69.6%). Table II provides the distributions in individual regions. In Östergötland, the median (range) age was 56 (1-113) years. In the other two regions (Jönköping and Kalmar), only age groups were documented. Prevalence of PALs differed with age and was lowest, at 0.5-0.7%, across the 3 regions in the youngest age group (0-12 years). The prevalence increased with age and was highest in the oldest age group (80+ years), at 5.4-7.5% (Fig 1). The proportion of children under 18 was 6.23% in Östergötland, 6.70% in Jönköping, and 4.89% in Kalmar.

**Table II:** Prevalence of PALs and clinics responsible for registration by Southeast Healthcare Region of Sweden

Characteristic	Total	Region		
		Östergötland	Jönköping	Kalmar
Prevalence of PALs in total	30,250 (2.8)	13,945 (2.9)	6,664 (1.8)	9,641 (3.9)
Patients with complex PALs	22,736 (75.1)	8,225 (58.9)	6,664 (100)	7,847 (81.3)
Female sex	21,081 (69.6)	9,628 (69.0)	4,635 (69.5)	6,818 (70.7)
Speciality registering label∗				
Primary care	6,110 (26.8)	2,286 (27.7)	1,666 (25.0)	2,158 (27.5)
Accident and emergency care	1,275 (5.6)	584 (7.1)	134 (2.0)	557 (7.0)
Allergy or dermatology clinic	807 (3.54)	230 (2.7)	499 (7.4)	78 (0.9)
Other	4,654 (20.4)	1,515 (18.4)	1,420 (21.3)	1,719 (21.9)
Unclear (mostly reregistered data from previous EMR systems)	9,890 (43.4)	3,610 (43.8)	2,945 (44.1)	3,335 (42.5)

Data are presented as nos. (%).

∗ Information available from complex cohorts only.

Phenotype analyses in the total cohorts of each region demonstrated considerable variation across regions. Between 24.4% and 64.7% of the labels lacked relevant information about symptoms (Table III). Skin symptoms were the most frequently reported across the 3 regions among labels with information about the index reaction (Table III). Mild, nonallergic adverse effects, primarily gastrointestinal, occurred in only 1.9-2.0% of the labels. Systemic reactions labeled *anaphylaxis* or described as a severe acute reaction with circulatory and/or respiratory compromise were recorded in 3.0-6.9% of cases.

**Table III:** Phenotypes of suspected hypersensitivity reactions to penicillin in Southeast Healthcare Region of Sweden

Characteristic	Total	Region		
		Östergötland	Jönköping	Kalmar
Individuals with PALs	30,250	13,945	6,664	9,641
Skin-related symptoms				
Urticaria and/or angioedema	7,286 (24.0)	3,112 (22.3)	1,066 (15.9)	3,108 (32.2)
Maculopapular drug eruption/nondefined rash	4,273 (14.1)	1,810 (12.9)	162 (2.4)	2,301 (23.8)
Exclusively pruritus	1,104 (3.6)	560 (4.0)	266 (3.9)	278 (2.8)
Flushing	1,832 (6.0)	692 (4.9)	401 (6.0)	739 (7.6)
Total	14,495 (47.9)	6,174 (44.2)	1,895 (28.4)	6,426 (66.6)
Anaphylaxis or breathing difficulties	1,418 (4.6)	420 (3.0)	328 (4.9)	670 (6.9)
Mild, nonallergic adverse effects (eg, diarrhea, nausea, headache)	599 (1.9)	285 (2.0)	128 (1.9)	186 (1.9)
Unclear symptoms	13,738 (45.4)	7,066 (50.6)	4,313 (64.7)	2,359 (24.4)

Data are presented as nos. (%).

Because the labels were systematically reregistered from the previous medical records over the decades before 2017, specific registration dates for labels before 2018 were not available. Registration dates after 2017 were accessible in the Jönköping region only, which enabled a recent time frame to be analyzed.

Narrow-spectrum penicillins (penicillin V and G) were the most commonly reported culprit in all regions, at 32.9-48.1%, followed by penicillins with extended spectrum (amoxicillin, ampicillin, pivmecillinam, piperacillin) in 8.5-15.4%, whereas flucloxacillin and cloxacillin were recorded in 3.9-6.2% (Table IV).

**Table IV:** Registered culprits in PALs

		Region			
Characteristic	Total of all 3 regions∗	Östergötland	Jönköping (total)	Jönköping (new)	Kalmar
Cohort	N = 22,025	Simple cohort only, n = 5,720	Total cohort, n = 6,664	New registrations since 2018 in total cohort,† n = 3,554	Total cohort, n = 9,641
Narrow-spectrum penicillins	9,462 (42.9)	1,887 (32.9)	2,931 (43.9)	1,197 (33.6)	4,644 (48.1)
Amoxicillin, ampicillin, piperacillin, or pivmecillinam	3,098 (14.0)	490 (8.5)	1,121 (16.8)	527 (14.85)	1,487 (15.4)
Cloxacillin or flucloxacillin	1,234 (5.6)	226 (3.9)	415 (6.2)	261 (7.3)	593 (6.1)
Unclear	8,231 (37.3)	3,117 (54.4)	2,197 (32.9)	1,569 (44.1)	2,917 (30.2)

Data are presented as nos. (%).

∗ Registered culprits from total cohorts of regions of Jönköping and Kalmar and simple cohort of Östergötland.

† Registration dates were only available in Jönköping, which yielded more precise analysis of newly registered labels since 2018. (Exact time frame for other registrations is uncertain thanks to systematic reregistering of old labels from 1980s).

To analyze the more recent pattern of culprits, labels recorded since 2018 in Jönköping (n = 3,554) were examined. Culprits were documented in 55.8% of the cases; narrow-spectrum penicillins accounted for 33.6% of labels, extended-spectrum penicillins for 14.8%, and cloxacillin and flucloxacillin for 7.3% of labels, suggesting that narrow-spectrum penicillins remain the most common culprits.

The identity of the providers who registered the labels was unclear in 42.5-44.1% of cases across all 3 regions. For the 12,846 patients whose data were available, primary care physicians registered 25.0-27.7% of the labels. Allergists or dermatologists were responsible for just 0.9-7.4% of registrations, reflecting significant regional variations in routines and resources (Table II).

Comparative analysis of the prevalence of multidrug-resistant bacteria, bloodborne diseases, and nonpenicillin drug allergy labels between the complex cohorts and the control groups showed a significantly higher prevalence of multidrug-resistant bacteria in patients with PALs compared to the control groups in all regions: 0.43 vs 0.24% in Östergötland (*P* = .03), 1.45 vs 0.30% (*P* < .001) in Jönköping, and 0.36 vs 0.19% (*P* = .049) in Kalmar. In Östergötland, the prevalence of bloodborne diseases was significantly higher than in the control group (0.27 vs 0.08%; *P* = .003), whereas no significant differences were found in the other two regions.

The prevalence of allergy labels to nonpenicillin drugs was significantly higher in patients carrying a PAL than in the control groups in all regions: 18.75 versus 4.06% in Östergötland, 23.97 versus 2.38% in Jönköping, and 25.56 versus 11.44% in Kalmar (*P* < .001; Table V).

**Table V:** Prevalence of multidrug-resistant bacteria, bloodborne disease, and nonpenicillin drug allergy labels in complex cohorts compared to respective control groups

Characteristic	Region of Östergötland∗				Region of Jönköping†				Region of Kalmar∗			
	Complex cohort	Control group	*P* value	RR (95% CI)	Complex cohort	Control group	*P* value	RR (95% CI)	Complex cohort	Control group	*P* value	RR (95% CI)
No. of subjects	8,225	8,234			6,664	6,570			7,847	7,103		
Multidrug-resistant bacteria	36	20	.03	1.80 (1.04, 3.11)	97	20	<.001	4.78 (2.96, 7.73)	29	14	.049	1.88 (0.99, 3.55)
Bloodborne disease	23	7	.003	3.29 (1.41, 7.66)	18	29	.098	0.61 (0.34, 1.10)	32	44	.069	0.66 (0.42, 1.04)
Non-PA labels	1,543	335	<.001	4.61 (4.11, 5.17)	1,598	157	<.001	10.03 (8.55, 11.78)	2,006	813	<.001	2.23 (2.07, 2.41)

Information on drug allergy labels, multidrug-resistant bacteria, and bloodborne infections was recorded in distinct sections of complex labels, enabling comparative analyses between complex cohorts and control groups. *CI,* confidence interval; *RR,* risk ratio.

∗ Complex cohort.

† Total cohort.

## Discussion

This is the first study to investigate the prevalence of PALs in Sweden across the entire Southeast Healthcare Region, encompassing the regions of Östergötland, Jönköping, and Kalmar, with a total of 1,088,000 inhabitants. The prevalence of PALs ranged 1.8-3.9% across the 3 regions, with 2.8% in the total cohort. This is notably lower than has been reported from epidemiologic studies in other countries.^4^, ^5^, ^6^, ^7^

Most studies in the literature have reported the prevalence of PALs or self-reported PA in hospitalized patients to range from 4.0% to 15.0%.^1^, ^2^, ^3^, ^4^, ^5^^,^^16^^,^^17^ Only a limited number of studies have investigated PAL prevalence in the general population, and these report lower prevalences (ranging 0.6-9.5%) than in hospitalized patients.^6^, ^7^, ^8^^,^^18^

There are no studies directly examining the prevalence of PALs in Sweden. Data from the Swedish Knee Arthroplasty Register shows that 10% of patients (n = 55,530) who underwent knee arthroplasty surgery for osteoarthritis were administered nonpenicillin antibiotics as preoperative prophylaxis, primarily due to documented or self-reported PA.^19^ Given that cloxacillin has been the first choice for perioperative antibiotics since the 1970s in Sweden, these data indirectly suggest that 10% of the cohort had a suspected PA. Because this is a selected cohort of surgical patients with a mean age of 69 years, and 57% women, findings from this study cannot be extrapolated to the general population.^19^

The prevalence of 2.8% PALs in the total cohort in this study is comparatively low. This might be explained by the strict policies on antimicrobial stewardship, for which Sweden is well recognized.^13^^,^^14^ Specifically, antibiotics are only available by prescription in Sweden, and prescription is guided by stringent criteria outlined in guidelines for treating most community-acquired infections.^13^^,^^14^ This results in lower antibiotic exposure than in many other countries.^20^^,^^21^ The Strama Network (Collaboration against Antibiotic Resistance in Sweden), which is a voluntary network of authorities and organizations at national level, is involved in implementing measures to reduce the use of antibiotics in primary health care.^22^^,^^23^

Local routines and collaboration between different professions also contribute to lower prevalence. Notably, Jönköping exhibited the lowest prevalence of recorded labels (1.8%). According to local practice, in 2010, when the current system was introduced, allergy specialists and pharmacists reviewed all labels for patients referred to the allergy clinic, regardless of the referral indication. Pharmacists and primary care physicians also collaborated to remove irrelevant labels, including those for mild nonallergic adverse effects. Primary care physicians also reviewed drug allergy labels for all individuals seeking care in their clinics, removing labels only relating to nonallergic adverse effects or in individuals who had tolerated the suspected culprit drug after a reaction. In 2017, after the software update, all existing PALs were systematically revised and updated to ensure consistency with the updated platform and documentation standards. According to current guidelines, pharmacists continue to play a consultative role, particularly for nonallergists, to ensure that only relevant information is recorded in drug allergy labels (personal communication with allergy specialist Patrik Nordenfelt, MD, PhD, and pharmacist Anne Hiselius).

The lower prevalence can also be attributed to current practice and guidelines in Sweden for managing patients with suspected PA, which significantly differ from international guidelines. One of the main differences is the interpretation of mild reactions limited to the skin, such as maculopapular or nonspecific exanthemas. Current Swedish guidelines advise against labeling if the phenotype is nonspecific exanthema without itching, so maculopapular rashes are not routinely documented under allergy labels.^24^, ^25^, ^26^ Although most cases of mild exanthema reactions can be delabeled after an allergy evaluation, it is crucial to recognize that some cases are triggered by immune-mediated reactions, which may be difficult to distinguish by history alone.^27^^,^^28^

Information about the symptoms was lacking in 24.4-64.7% of the cases across the 3 regions, reflecting varying degrees of consistency and potentially compromising data validity. The high proportion of inconsistent labels suggests that awareness of PA and its potential negative consequences need to be strengthened. Despite these inconsistencies, the available data still allowed us to identify important patterns. Our findings indicate that serious allergic reactions were rarely documented, at 3.0-6.9% across the 3 regions. Moreover, cutaneous manifestations were the most common phenotypes in PALs across all cohorts (28.4-66.6%). The prevalence of primary skin manifestations, such as urticaria and maculopapular exanthema, varied significantly. The differences in the reported prevalence of skin manifestations and severe systemic reactions suggest that the interpretation and definitions differ among physicians, especially in primary care and acute and emergency settings, where most allergy labels are recorded.

Accurate population-based data on the prevalence of the different manifestations of PA are limited in the literature. A retrospective analysis of reported PA in an urban outpatient population in adult patients in an internal medicine clinic in New York showed that most cases (71.6%) were labeled because of skin symptoms.^29^ Given that 20.2% of the labels were unclear, 90% of the well-documented labels were entered because of a reaction in the skin, which is in line with our findings. The study also reported that anaphylaxis and breathing difficulties accounted for 6.8% and 3.4% of the labels, respectively. The prevalences are somewhat higher than in our study, in which the combined prevalence of anaphylaxis and breathing difficulties together was 3.0-6.9%. This difference should be interpreted with care because data from all age groups were analyzed in our study; the proportion of individuals under 18 was 6.2% in Östergötland, 6.7% in Jönköping, and 4.9% in Kalmar. The prevalence of severe systemic reactions due to PA in children is lower than in adults.^29^

Analysis of the culprits revealed that narrow-spectrum penicillins (penicillins V and G) were the most prevalent culprits across all cohorts over time. This pattern was observed in the earlier cohorts and in a more recent cohort consisting of cases in 3,554 patients that have been labeled since 2018. These findings are in line with the 2022 Global Antimicrobial Resistance and Use Surveillance System (aka GLASS) report, which indicated that the use of narrow-spectrum penicillins is still more frequent in Sweden and Scandinavia than in other countries.^30^

This specifically Scandinavian finding can be attributed to restrictive antibiotic policies and continued focus on antimicrobial stewardship, which advocate the use of narrow-spectrum penicillins as first-line antibiotics in most community-acquired infections. A European Surveillance of Antimicrobial Consumption (aka ESAC) project that scrutinized penicillin usage across 25 countries in Europe revealed that narrow-spectrum penicillin (primarily phenoxymethylpenicillin) accounted for over 60% of penicillin use in Scandinavia, in contrast with less than 2% in the Mediterranean region. In most non-Scandinavian countries, penicillins with extended spectrum (primarily amoxicillin) were the most commonly prescribed. In 2003, combinations of penicillins with β-lactamase inhibitors represented more than 50% of penicillin use in Central and Southern Europe.^31^

Previous studies have indicated that drug allergies in general, but most notably PALs, are associated with increased prevalence of *Clostridium difficile,* methicillin-resistant *Staphylococcus aureus,* and vancomycin-resistant *Enterococcus*.^9^ Additionally, scrutiny of the impact of a reported PA in a surgical setting suggested that carrying a PAL increased the odds of developing a surgical site infection by 50% because of the use of non–β-lactam antibiotics for prevention of surgical site infection.^10^ In the present study, the prevalence of multidrug-resistant bacteria was significantly higher across the 3 complex cohorts compared to the control groups. These findings are in line with previous studies, indicating that carrying a PAL is associated with an increased risk of multidrug-resistant bacteria, likely as a result of the overuse of broad-spectrum antibiotics in individuals with suspected PA.^9^^,^^10^ Furthermore, a significantly higher prevalence of nonpenicillin drug allergy labels was observed in the complex cohorts of patients with a PAL compared to control groups (Table V).

Although our findings are based on data from 3 Scandinavian regions, false PA labeling and antibiotic overuse leading to multidrug resistance represent major challenges worldwide. Because a significant part of the PALs are recorded by nonallergists, greater collaboration between allergists and nonallergists should be facilitated by developing guidelines and protocols. Our findings suggest that allergy labels can be more accurately documented by educating nonallergists about the main underlying mechanisms of allergic reactions, the main cutaneous manifestations of drug allergy, anaphylaxis criteria, and allergic versus nonallergic reactions. More precise documentation, such as photographs of skin symptoms, detailed history taking regarding symptom description and their duration, chronology, treatment, subsequent exposure to the culprit or potentially cross-reactive drugs after the index reaction, monitoring vital signs, and measuring serum tryptase in suspected anaphylactic cases will enable allergists to better assess patients with PALs. This is particularly important considering that urticaria and maculopapular exanthema represent different immunologic pathways and require distinct evaluation methods (eg, skin tests, specific IgE tests, and types of provocation) in line with current international guidelines.^27^^,^^28^ Education and training for physicians, particularly in primary care, where most labels are entered, could improve both the quality of documentation and awareness of the clinical consequences of inaccurate labeling. Timely referrals and greater involvement of nonallergists in assessing suspected penicillin allergies, such as delabeling low-risk patients, will improve evaluation efficiency and mitigate the adverse effects of false labeling.

Considering the discrepancies between Swedish and international guidelines concerning the interpretation of allergic reactions to penicillin, it is crucial to align with international standards in this field. This will ensure that all patients are assessed using safe, evidence-based methods. Additionally, involving nonallergist physicians, such as infectious medicine specialists and microbiologists, will facilitate delabeling and tackle the overuse of broad-spectrum antibiotics.^2^^,^^32^^,^^33^

Our results support that careful evaluation to avoid incorrect PALs could reduce the risk of developing multidrug-resistant bacteria and mitigate further negative consequences associated with the PALs.

There are limitations to this study. First, some PALs may not have been identified using our search methods. To calculate the total prevalence of PALs, it was necessary to include nonupdated simple labels. These simple labels lacked standardized documentation and ATC codes, and only free-text search could be used to identify them. Although we attempted to improve sensitivity by including truncations, alternative abbreviations, and trade names of current and discontinued penicillin-containing medications, the method has not been validated in smaller cohorts. Additionally, the descriptions of index reactions may not always be accurate. Moreover, the simple and complex cohorts were not fully comparable; in two regions, a substantial proportion of allergy labels was documented only as “simple labels,” corresponding to the simple cohorts. Because this study aimed to analyze the prevalence of PALs across 3 Swedish regions, even nonupdated labels were included. Additionally, in Östergötland, information on suspected culprit drugs was available only in the simple cohort. To characterize phenotypes across all 3 cohorts, we considered it important to comprehensively analyze the simple cohorts to better understand how suspected penicillin allergies are documented. A further limitation is that labeling for multidrug-resistant bacteria was recorded only as a binary yes/no variable during data extraction, and we did not have access to complete EMRs or to electronic laboratory data on microbiologic samples and cultures. As a result, we may have missed patients with multidrug-resistant bacteria and bloodborne diseases. Furthermore, control groups and complex cohorts were not matched to each other with respect to comorbidities, and for the analysis concerning multidrug-resistant bacteria, bloodborne disease, and nonpenicillin drug allergy labels, such matching would likely allow for a more appropriate comparison.

In conclusion, our results suggest that the PAL prevalence is low in the Southeast Healthcare Region of Sweden for several reasons. The observed low prevalence may be explained by the restrictive antibiotic policy and continued focus on antimicrobial stewardship. In up to 65% of cases, PALs lack adequate information. Therefore, more accurate documentation is necessary to appropriately manage patients with suspected PA. In the future, to improve the quality of the data in future studies, it will be necessary to gain access to complete EMRs, rather than labels only. Moreover, search methods may need to be verified.

## Disclosure statement

Financial support was received from the Swedish Asthma and Allergy Association’s Research Fund and the 10.13039/501100000265Medical Research Council of Southeast Sweden.

Disclosure of potential conflict of interest: The authors declare that they have no relevant conflicts of interest.
