# Supplementary material for: Epidemiology of penicillin allergy labels in a general Swedish population
Source: J Allergy Clin Immunol Glob. 2026 Jul 17;5(5):100761. doi: 10.1016/j.jacig.2026.100761 (PMC13472025; doi:10.1016/j.jacig.2026.100761)
Supplement: Supplementary file 1 [file mmc1.docx]

**Supplementary information**

**Healthcare information systems in the Southeast Healthcare Region**

Most healthcare in Sweden is provided by public health services, in which various health care information systems are operated to store, manage, and transmit electronic medical records. In the Southeast Healthcare Region, a unified program is used to maintain medical records. During the study period, each individual’s health records included an electronic icon with details about drug allergies, multiresistant bacteria, bloodborne infections, transplantation history, and other vital information, all of which is available to healthcare professionals. Penicillin allergy labels (PALs) can be entered by any physician if a patient has a suspected or confirmed penicillin allergy. Only physicians at each level of care are authorized to add or remove drug allergy labels, and a suspicion of allergy is generally considered sufficient for documenting it. Once recorded, the label remains in the EMR until a physician manually reviews and removes it.

Historically, different methods have been used to register suspected drug allergies. Before 2017, suspected drug allergies were recorded in free text, resulting in simple drug allergy labels consisting of two headings: a title and a comment, without specific requirements concerning details of the allergy. For individuals carrying a penicillin allergy label while paper-based or electronic healthcare systems were used, the relevant information has been consistently re-recorded following the implementation of a new system.

Consequently, labels recorded before 2017 display significantly varying documentation dates and do not refer to the initial reaction date in each case. A software update in 2017 introduced a system that required detailed information about suspected drug allergies, including symptoms and Anatomical Therapeutic Chemical code (ATC-code) for the pertinent medication or group of medications. Additionally, suspected hypersensitivity to food or other substances, bloodborne infections, multidrug-resistant bacteria, etc., could be recorded under complex labels. The goal was for healthcare providers to revise simple labels and upgrade them to complex allergy labels or clear them if no longer relevant.

The exact dates of the cohort reviews are unknown. The new program that enabled the caregivers to register the complex labels was introduced during the Fall of 2017 across the healthcare region. Labels began successively to be revised after the introduction of the program.

**Identification of the cohorts**

Data were extracted according to the inclusion criteria following the same general methodology across regions; however, depending on regional ethical approvals, certain data were aggregated for specific variables. All data were anonymized, and the variables were provided to the researchers in Microsoft Excel files.

IT technicians identified individuals with penicillin allergy labels in the respective regions using two different methods. The simple cohorts were identified using keywords associated with penicillin allergy (e.g., “pc”, “cillin” various substance names, and trade names) that were documented under the labels as a header or a comment. As it had limited specificity, the search method led to non-relevant labels being retrieved, such as those for APC resistance or pacemakers, which required manual review and correction. Moreover, some PALs may not have been identified using this method. Therefore, we enhanced sensitivity by including truncations, alternative abbreviations, and trade names of current and discontinued penicillin-containing medications. The method has not been validated in smaller cohorts. The complex cohorts were created using the ATC codes for penicillin-containing medications. This method enabled more accurate identification of penicillin allergy labels, thereby eliminating the need of further review for this cohort.

Control groups were also established by the IT technicians according to additional criteria: randomly selected individuals not carrying a penicillin allergy label. The proportions of age groups, sexes and municipalities were matched to the complex cohorts to align the size of the control groups with the complex cohorts at a ratio of 1:1. Given that the initial data incorporated variables from deceased persons, data related to these individuals were manually reviewed and removed. As a result, the control groups do not exactly match the complex cohorts 1:1.

**Documentation of symptoms in the different cohorts**

Simple cohorts

In the simple cohorts, symptom descriptions were recorded as free text and identified via keywords. During the data analysis, common manifestations of drug allergy reactions, such as medical terms in Latin and Swedish, were used as keywords, with some truncated to avoid overlooking relevant records due to potential misspellings (e.g., “urti”, “anaph”, “anaf”, “exant”, etc.). If a reaction was described with multiple keywords, it was classified under the most severe manifestation. For instance, a reaction characterized by hypotension and urticaria was documented solely as “anaphylaxis,” whereas a reaction described as urticaria, edema, and itching was categorized as “urticaria with angioedema.”

**Complex cohorts**

Symptoms were recorded in two distinct manners: whereas some labels were entered as free text, most symptom descriptions were standardized into categories such as “severe urticaria”, “mild to moderate urticaria”, etc.

Culprits

In the Region of Östergötland, culprits were available in the simple cohort only and were specified in 2,603 out of 5,720 (45.5%) cases. Narrow-spectrum penicillins (penicillin V and G) were the most prevalent, appearing in 32.9% of the simple cohort. Penicillins with extended spectrum (amoxicillin, ampicillin, pivmecillinam, piperacillin) were documented in 8.5% of cases, with flucloxacillin recorded in 3.9% (Table 4).

In Jönköping and Kalmar region’s complex cohorts, culprits were registered under specific ATC codes, whereas in Kalmar’s simple cohort they were documented as free-text entries. However, the precise culprit remained unclear in 30.2–32.9% of the labels.

In the Region of Jönköping, specific ATC codes were registered for 67.0% (n=4,467) of the total cohort (Table 4). Narrow-spectrum penicillins accounted for 43.9% of the cases, and penicillins with extended spectrum for 16.8%. To assess more recent patterns in this region, labels recorded since 2018 were analyzed (n=3,554). Culprits were documented in 55.8% of cases, showing a similar distribution: narrow-spectrum penicillins remained the most common (33.6%), followed by extended-spectrum penicillins (14.8%), with cloxacillin and flucloxacillin representing 7.3%.

In the Region of Kalmar, the culprits were recorded in 69.7% (n=6,724) of the total cohort.

Narrow-spectrum penicillins accounted for 48.1% of the cohort. Penicillins with extended spectrum were recorded in 15.4% of cases. Cloxacillin and flucloxacillin were noted in 6.1% (Table 4).
